# LIN28A immunoreactivity is a potent diagnostic marker of embryonal tumor with multilayered rosettes (ETMR)

**DOI:** 10.1007/s00401-012-1068-3

**Published:** 2012-11-16

**Authors:** Andrey Korshunov, Marina Ryzhova, David T. W. Jones, Paul A. Northcott, Peter van Sluis, Richard Volckmann, Jan Koster, Rogier Versteeg, Cynthia Cowdrey, Arie Perry, Daniel Picard, Marc Rosenblum, Felice Giangaspero, Eleonora Aronica, Ulrich Schüller, Martin Hasselblatt, V. Peter Collins, Andreas von Deimling, Peter Lichter, Annie Huang, Stefan M. Pfister, Marcel Kool

**Affiliations:** 1 Clinical Cooperation Unit Neuropathology, German Cancer Research Center (DKFZ), Im Neuenheimer Feld 280, Heidelberg, Germany; 2 Department of Neuropathology, NN Burdenko Neurosurgical Institute, 4th Tverskaya-Yamskaya 16, Moscow, 125047 Russia; 3 Division of Pediatric Neurooncology, German Cancer Research Center (DKFZ), Im Neuenheimer Feld 580, 69120 Heidelberg Germany; 4 Department of Oncogenomics, Academic Medical Center, Amsterdam, The Netherlands; 5 Departments of Pathology and Neurological Surgery, Brain Tumor Research Center, University of California, San Francisco, USA; 6 Division of Hematology-Oncology, Department of Pediatrics, Arthur and Sonia Labatt Brain Tumour Research Centre, Hospital for Sick Children, University of Toronto, Toronto, ON Canada; 7 Department of Pathology, Memorial Sloan-Kettering Cancer Center, New York, USA; 8 Department of Radiological, Oncological and Anatomic Pathology Sciences, Università Sapienza, Rome, Italy; 9 IRCCS Neuromed, Pozzilli, Italy; 10 Department of Neuropathology, Academic Medical Center, Amsterdam, The Netherlands; 11 Center of Neuropathology, Ludwig-Maximilians University, Munich, Germany; 12 Institute of Neuropathology, University Hospital Münster, Münster, Germany; 13 Department of Pathology, University of Cambridge, London, UK; 14 Divison of Molecular Genetics, German Cancer Research Center (DKFZ), Heidelberg, Germany; 15 Department of Pediatric Hematology and Oncology, Heidelberg University Hospital, Heidelberg, Germany; 20 Department of Neuropathology, Heidelberg University Hospital, Heidelberg, Germany

**Keywords:** ETMR, Pediatric brain tumor, LIN28A, Diagnostic marker

## Abstract

Embryonal tumor with multilayered rosettes (ETMR, previously known as ETANTR) is a highly aggressive embryonal CNS tumor, which almost exclusively affects infants and is associated with a dismal prognosis. Accurate diagnosis is of critical clinical importance because of its poor response to current treatment protocols and its distinct biology. Amplification of the miRNA cluster at 19q13.42 has been identified previously as a genetic hallmark for ETMR, but an immunohistochemistry-based assay for clinical routine diagnostics [such as INI-1 for atypical teratoid rhabdoid tumor (AT/RT)] is still lacking. In this study, we screened for an ETMR-specific marker using a gene-expression profiling dataset of more than 1,400 brain tumors and identified LIN28A as a highly specific marker for ETMR. The encoded protein binds small RNA and has been implicated in stem cell pluripotency, metabolism and tumorigenesis. Using an LIN28A specific antibody, we carried out immunohistochemical analysis of LIN28A in more than 800 childhood brain-tumor samples and confirmed its high specificity for ETMR. Strong LIN28A immunoexpression was found in all 37 ETMR samples tested, whereas focal reactivity was only present in a small (6/50) proportion of AT/RT samples. All other pediatric brain tumors were completely LIN28A-negative. In summary, we established LIN28A immunohistochemistry as a highly sensitive and specific, rapid, inexpensive diagnostic tool for routine pathological verification of ETMR.

## Introduction

Embryonal tumor with multilayered rosettes (ETMR), also previously reported as embryonal tumor with abundant neuropil and true rosettes (ETANTR) is a rare, albeit likely underdiagnosed embryonal CNS neoplasm with fewer than 100 cases reported in the literature since its initial description in 2000 [3, 5, 19]. In the 2007 WHO classification, ETANTR was only discussed as a possibly unique variant of CNS tumors [14]. Supratentorial localization has mainly been reported, but it may also occur in the brain stem and cerebellum [3, 5, 11]. ETMR predominantly affects children under the age of 3–4 years and is associated with a highly aggressive disease course with reported overall survival times ranging from 5 to 30 months. The histopathological appearance is distinctive in its mixture of poorly differentiated small cell areas with prominent multilayered (ependymoblastic) rosettes, and areas with focal neuronal differentiation, thus combining features of CNS neuroblastoma and ependymoblastoma [3, 5, 11]. On the other hand, a wide spectrum of morphological patterns may be encountered, with some features being considerably less specific. For this reason, misdiagnosis is not uncommon, with common considerations including “small round blue cell tumor, not otherwise specified”, i.e., central nervous system primitive neuroectodermal tumors (CNS PNET) including controversial variants (e.g., ependymoblastoma, central neuroblastoma), medulloblastoma, and anaplastic ependymoma [9, 13, 25]. Moreover, similar rosettes can sometimes occur in other embryonal CNS tumors (e.g., CNS PNET, AT/RT) [7]. Nevertheless, accurate pathological verification of ETMR is of important clinical relevance, given its poor response to current CNS PNET treatment protocols and thus universally fatal outcome [5, 11, 13]. Amplification of a miRNA cluster at 19q13.42 has been identified as a genetic hallmark of ETMR, affecting up to 95 % of samples tested and it is considered a unifying molecular diagnostic marker for these tumors [11, 13, 16, 25].

However, the requirement of non-commercial custom made BAC probes for FISH testing renders this assay virtually inaccessible to nearly every pathology laboratory around the globe. In contrast, the use of immunohistochemistry is universally utilized in neuropathology centers everywhere. As such, ETMR is a highly attractive target for developing tumor-specific immunohistochemistry. For this purpose, we herein performed a screen for ETMR-specific genes based on gene expression profiles collected for a large cohort of various brain tumors (*n* = 1,404). We selected the most specifically expressed gene, namely *LIN28A*, and subsequently tested its diagnostic specificity for ETMR with a high-quality commercial antibody on a large cohort (*n* = 816) of paraffin-embedded brain tumors. Our results show that LIN28A expression can be used as a specific diagnostic biomarker to detect ETMR.

## Materials and methods

To find ETMR specific marker genes, we used 849 publicly available gene expression profiles [1, 4, 6, 8, 10, 17, 18, 21, 23] plus 555 newly generated profiles (total 1,404) of primary pediatric and adult brain tumors including low-grade glioma (LGG; *n* = 7), pilocytic astrocytoma (PA; *n* = 129), ependymoma (EP; *n* = 162), astrocytoma grade II (AII; *n* = 22), oligodendroglioma grade II (OII; *n* = 47), oligoastrocytoma grade II (OAII; *n* = 3), anaplastic astrocytoma grade III (AAIII; *n* = 49), anaplastic oligodendroglioma grade III (AOIII; *n* = 60), anaplastic oligoastrocytoma grade III (AOAIII; *n* = 25), glioblastoma grade IV (GBM; *n* = 349), diffuse infiltrating pontine glioma (DIPG; *n* = 29), CNS PNET (*n* = 43), atypical teratoid rhabdoid tumor (AT/RT; *n* = 37), medulloblastoma (MB; *n* = 429), and ETMR (*n* = 13). All gene-expression profiles were generated by Affymetrix U133plus 2.0 arrays using RNA isolated from frozen tumors. For comparison we used the publicly available gene expression profiles of 353 normal CNS and non-CNS tissues [22]. All data were analysed using the microarray analysis and visualization platform R2 (http://r2.amc.nl). For immunohistochemical (IHC) analysis of LIN28A, a polyclonal antibody (A177, #3978, Cell Signaling Inc, Boston, MA, USA) was applied to a partly overlapping (~10 % of the cases was also included in the expression profiling) cohort of 816 malignant pediatric brain tumors constructed on tissue microarrays (patient age <18 years; see Table 1), including MB (*n* = 334); EPIII (*n* = 223); GBM (*n* = 131); AT/RT (*n* = 50); CNS PNET (*n* = 41); ETMR (*n* = 37). All cases, except for a few ETMR cases contributed by collaborators, were obtained from the NN Burdenko Neuropathological Institute (Moscow, Russia). IHC was performed with an automated stainer (Benchmark; Ventana XT) using antigen-retrieval protocol CC1 and a working antibody dilution of 1:100 with incubation at 37 °C for 32 min. In parallel, FISH analysis for the 19q13.42 locus was performed for all tumors as previously described [11].

**Table 1:** Distribution of LIN28A immunoexpression in various pediatric brain tumors

Tumor type	*n*	LIN28A + diffuse	LIN28A + focal	INI1 negativity	19q13.42 amplification
ETMR	37	37 (100 %)	0	0	36 (97 %)
AT/RT	50	0	6 (12 %)	50 (100 %)	0
stPNET	41	0	0	0	0
MB	334	0	0	0	0
EPIII	223	0	0	0	0
GBM	131	0	0	0	0

## Results and discussion

**Fig. 1:**
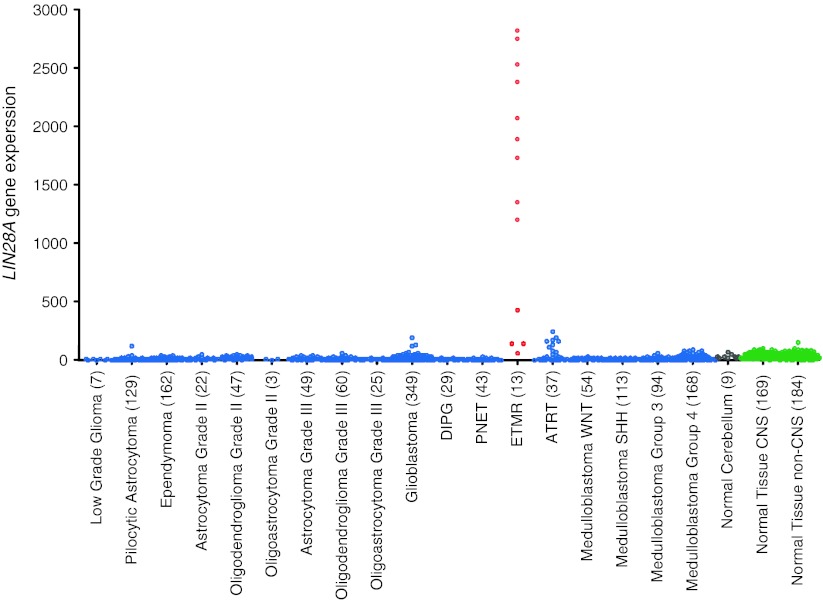
*LIN28A* expression is highly upregulated only in ETMR cases when compared to other pediatric and adult brain tumors (*n* = 1404) and normal CNS and non-CNS tissues (*n* = 353). The *numbers in parentheses* indicate the number of samples for each entity. All data were generated on Affymetrix U133plus 2.0 arrays and were analysed using the microarray analysis and visualization platform R2 (http://r2.amc.nl)

As an initial step, a comparative supervised analysis of gene-expression profiles generated for a large cohort of various pediatric and adult brain tumors was performed. When comparing the 13 ETMR samples with all other brain tumors, we identified a specific gene signature that could readily distinguish ETMR samples from all other brain tumors. Among the 50 most differentially expressed genes, 18 (*LIN28A*, *IGF2BP1*, *CRYBA1*, *HIC2*, *PRPF31*, *SALL4*, *SHISA3*, *HMGA2*, *LOC440416*, *PRTG*, *LOC253842*, *DNMT3B*, *CRABP1*, *C2ORF29*, *MRPS9*, *FGFBP3*, *SP8*, and *HES5*) were highly upregulated in all ETMR samples. However, only one gene, *LIN28A*, was highly upregulated in ETMR but not in any other brain tumor (Fig. 1). Nine ETMR samples showed particularly high LIN28A expression (>1,000). For all other brain tumors, only a subset of AT/RTs and a few other tumors showed low *LIN28A* mRNA levels, but otherwise, it was not expressed at all in other pediatric and adult brain tumors. Normal CNS and non-CNS tissues also did not express *LIN28A*. These data strongly suggest that *LIN28A* expression could be exploited as a diagnostic biomarker to specifically detect ETMR. Interestingly, Picard et al. [20] recently analysed gene-expression profiles of 51 CNS PNETs and identified 3 molecular subgroups, one of which was distinguished by high expression of *LIN28A*, frequent amplification of 19q13.42, young age, and dismal prognosis. Our data now suggest that all tumors in this subgroup fit well to the diagnosis of ETMR.

**Fig. 2:**
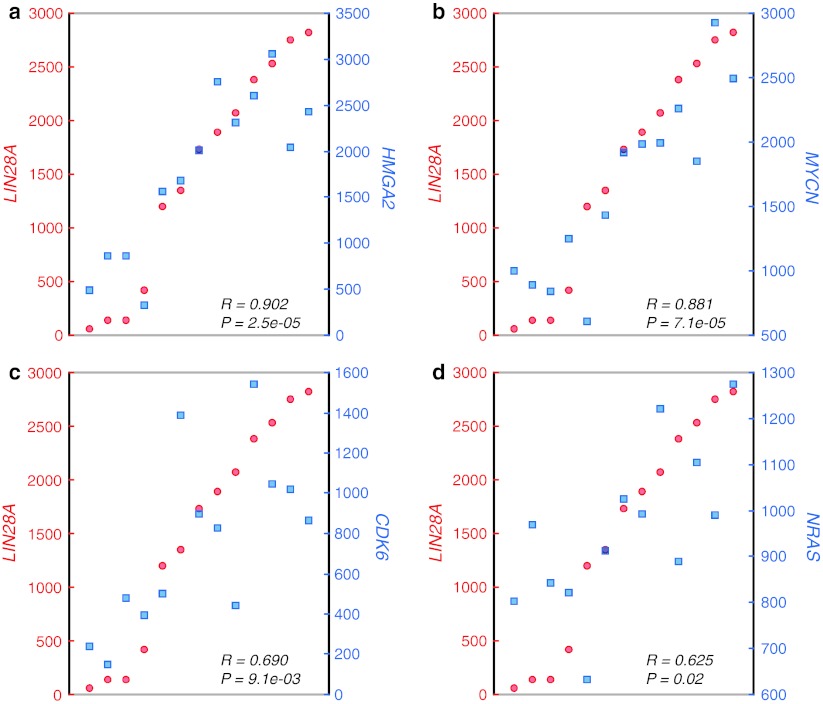
Expression of *LIN28A* strongly and significantly correlates with expression of *HMGA2* (**a**), *MYCN* (**b**), *CDK6* (**c**), and *NRAS* in ETMR (**d**)

*LIN28A* and its homolog *LIN28B* encode proteins that bind small RNA and function as negative regulators of the *let*-*7* family of miRNAs, which may act as tumor suppressor miRNAs [24]. LIN28A is a conserved cytoplasmic protein, but may be imported to the nucleus where it regulates the translation and stability of mRNA. In addition, LIN28A has been implicated in stem cell pluripotency and metabolism, is expressed widely in early embryogenesis, and is downregulated upon differentiation [7, 15]. Some recent studies suggest that *LIN28A* and *LIN28B* function as oncogenes promoting tumor transformation and progression, with high expression associated with unfavorable clinical course in cancers of the ovary, colon, esophagus, and sympathetic nervous system (neuroblastoma) [7, 15]. A potential mechanism for the oncogenic potency of LIN28A and LIN28B might be mediated through *let*-*7* repression and consecutive upregulation or stabilization of *let*-*7* targets, such as *MYC(N)*, *RAS*, *CDK6* and *HMGA2* [12]. Of note, we indeed observed strikingly tight associations between expression levels of *LIN28A* and those of *MYCN*, *NRAS*, *CDK6*, and *HMGA2* in the 13 ETMRs (Fig. 2). Moreover, recent data of Molenaar et al. [15] showed that *Lin28B* overexpression in the mouse sympathetic adrenergic lineage induced development of neuroblastomas marked by low *let*-*7* miRNA and high MYCN expression. Interestingly, *LIN28B* is also highly expressed in all 13 ETMRs, but its diagnostic utility is limited, given that it is also found in other brain tumors, including medulloblastoma.

**Fig. 3:**
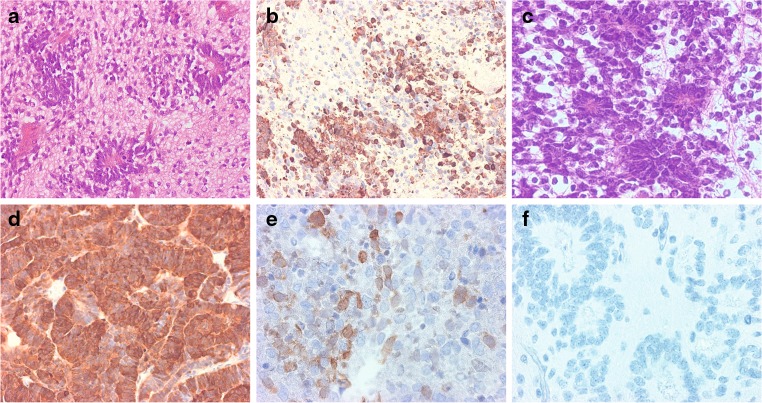
LIN28A immunohistochemistry in pediatric malignant CNS tumors. Microscopic appearance of ETMR composed of clusters of multilayered rosettes embedded in abundant neuropil (initially diagnosed as ETANTR) (**a**). Intense LIN28A expression in poorly differentiated areas containing rosettes and the absence of expression in neuropil of tumor (**b**). Microscopic appearance of ETMR composed of numerous rosettes with clusters of small poorly differentiated cells (initially diagnosed as ependymoblastoma) (**c**). Strong and diffuse LIN28 expression in all parts of the tumor (**d**). AT/RT. Focal LIN28A expression in single collections of tumor cells (**e**). Anaplastic ependymoma. Immunonegativity for LIN28A in tumor region with numerous ependymal rosettes (**f**)

**Fig. 4:**
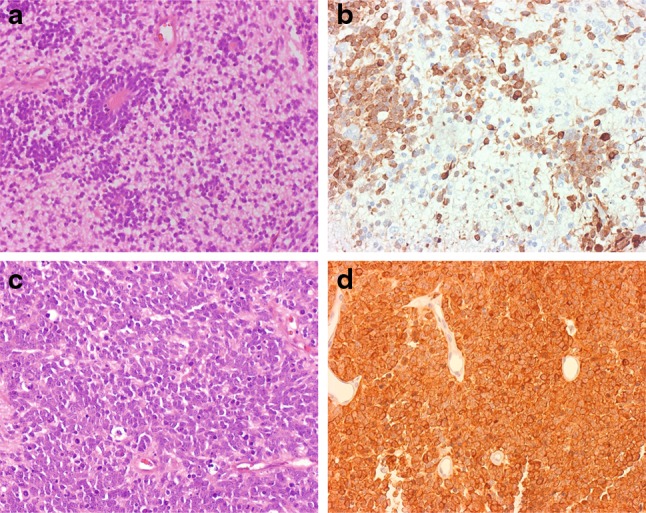
LIN28A expression patterns in primary and recurrent ETMR. Primary ETMR with single multilayered rosettes and abundant neuropil (**a**). LIN28A expression is limited with rosettes and small cell collections (**b**). The ETMR relapse from the same patient is composed of hypercellular areas but lacks neuropil areas (**c**). Strong and diffuse LIN28A expression in all cells of the recurrent tumor (**d**)

To test whether LIN28A expression could be used as a sensitive and specific diagnostic marker for ETMR, we performed IHC screening of a large cohort of 816 malignant pediatric brain tumors (Table 1). Strong and diffuse LIN28A cytoplasmic immunostaining was found in all 37 histologically classic ETMR (Fig. 3). Thirty-six of these tumors (97 %) also harbored high-level amplification of the 19q13.42 locus, which was not present in any other tumors analyzed (Table 1). LIN28A positivity was found to be more prominent and intense in multilayered rosettes and poorly differentiated small cell tumor areas, whereas only single collections of positive cells were observed within neuropil-like tumor parts. Analysis of nine ETMR samples obtained from corresponding tumor recurrences showed that the intensity of LIN28A immunoexpression and the number of stained cells were significantly higher in recurrent lesions in comparison to their primaries (Fig. 4). In addition, we had the opportunity to compare the results of LIN28A mRNA and protein expression for six ETMR samples (4 tumors with expression gene levels >1,000, and 2 samples with lower LIN28A expression). All these tumors showed clear LIN28A positivity. Nevertheless, the two ETMR samples with lower *LIN28A* mRNA expression levels contained extensive areas of LIN28A immunonegative neuropil, whereas the four tumors with high *LIN28A* mRNA expression were composed predominantly of LIN28A immunopositive, histologically primitive small cell areas.

**Fig. 5:**
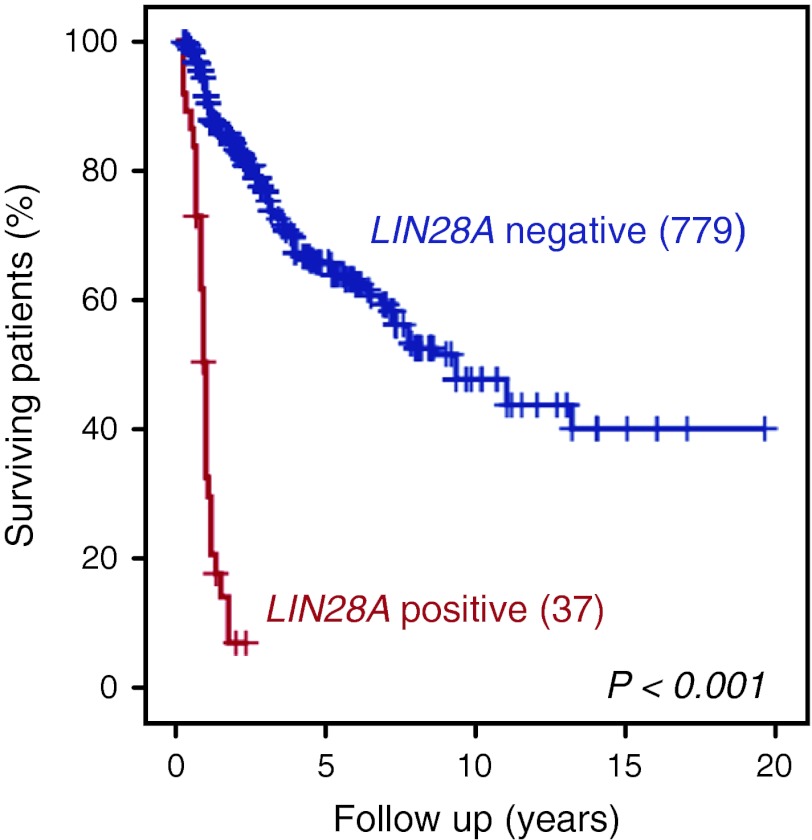
Kaplan-Meier overall survival analysis for LIN28A positive malignant brain tumors versus LIN28A negative malignant brain tumors in children. Overall 5-year survival was 0 % for LIN28A positive versus 68 % for LIN28A negative tumors (log-rank,* p* < 0.001). Numbers on the Y-axis indicate the fraction of surviving patients. *Numbers on the X axis* indicate the follow-up time in years

Among the other tumor entities, focal cytoplasmic immunoexpression of LIN28A was only found in single cell collections of six AT/RT cases (12 %). Each of these six tumors had obvious losses of nuclear INI1 protein expression, in line with their initial histopathological diagnosis (Fig. 3). In contrast, all other pediatric CNS malignant tumors, including 44 additional AT/RT samples, 41 CNS PNETs, 334 medulloblastomas, 223 anaplastic ependymomas, and 131 pediatric glioblastomas were completely negative for LIN28A immunostaining (Fig. 3). Finally, we performed survival analysis for all these patients based on LIN28A reactivity (Fig. 5). Tumors with diffuse and wide-spread immunoexpression of LIN28A (considered as “positive” samples and representing all ETMR cases) showed an extremely poor overall survival (5-year overall survival estimate of 0 %) in comparison to other malignant pediatric brain tumors with absence or rare focal positivity of LIN28A (5-year survival 68 %, log-rank *p* < 0.001).

Therefore, this study represents a report of LIN28A as a highly specific and sensitive diagnostic marker for the distinct pathological verification of ETMR and, correspondingly, as a new important diagnostic tool to be routinely applied in all malignant childhood brain tumors where ETMR may be considered a possible diagnosis. Variability of *LIN28A* gene expression in ETMR samples correlated closely with the intratumoral heterogeneity observed immunohistochemically. Focal positivity of LIN28A in a small subset of AT/RTs is not considered problematic when taking the simultaneous loss of INI1 expression into account, with the latter representing a fairly specific molecular marker for AT/RT. On the other hand, LIN28A immunoexpression has also been detected recently in CNS germ-cell tumors with diffuse staining patterns found in germinomas, embryonal carcinomas and yolk sac tumors but being only focal in immature teratomas [2]. Nevertheless, these tumors usually affect older children (apart from the some immature teratomas of infancy) than those at risk for ETMRs, are typically not situated in those locations commonly involved by ETMRs, and do not enter the differential diagnosis on histopathologic and immunohistochemical grounds.

Our current data also confirmed that ETMR is a unique clinico-pathologic entity from a molecular perspective, exhibiting 19q13.42 amplification and high levels of LIN28A expression accompanied by particularly aggressive clinical behavior. It will now be important to understand the biological consequence of overexpression of such universal and direct miRNA translation regulators as LIN28A and LIN28B in ETMR, taking into account the prototypic amplification of the oncogenic miRNA cluster at 19q13.42 in these tumors.
